# DSAIL-Porini: Annotated camera trap image data of wildlife species from a conservancy in Kenya

**DOI:** 10.1016/j.dib.2022.108863

**Published:** 2022-12-27

**Authors:** Lorna Mugambi, Jason N. Kabi, Gabriel Kiarie, Ciira wa Maina

**Affiliations:** a Centre for Data Science and Artificial Intelligence (DSAIL), Dedan Kimathi University of Technology, Private Bag-10143, Nyeri, Kenya; b Department of Electrical and Electronic Engineering, Dedan Kimathi University of Technology, Private Bag-10143, Nyeri, Kenya

**Keywords:** Image classification, Object detection, Machine learning, Ecological data

## Abstract

For years, zoologists, ecologists, and researchers at large have been using instruments such as camera traps in acquiring images of wild animals non-intrusively for ecological research. The main reason behind ecological research is to increase the understanding of various interactions in ecosystems while providing supporting data and information. Due to climate change and the destruction of animal habitats in recent years, researchers have been conducting studies on diminishing populations of various species of interest and the effectiveness of habitat restoration practices. By collecting and examining wild animal image data, inferences such as the health, breeding rate, and population of a particular species can be made. This paper presents an annotated camera trap dataset, DSAIL-Porini^1^, consisting of images of wildlife species captured in a conservancy in Nyeri, Kenya. 6 wildlife species are captured in this dataset: impala, bushbuck, Sykes’ monkey, defassa waterbuck, common warthog, and Burchell's zebra. This dataset was collected using camera traps based on the Raspberry Pi 2, Raspberry Pi Zero, and OpenMV Cam H7. It provides an example of images collected using relatively low-cost hardware platforms. The image dataset can be used in training and testing object detection and classification machine learning models.

**The Specifications Table**

**Table:** 

Subject	Environmental Science
Specific subject area	Ecology
Type of data	Images
How the data were acquired	The data were acquired using camera traps deployed in a wildlife conservancy during the day. Annotation of the images was done including; species in an image, the count, sex of the animals, and the coordinates of the camera trap for each image captured.
Data format	Raw, jpg
Description of data collection	Programing Language: Python Sensor: Passive infrared sensor (PIR) Cameras used: Raspberry Pi camera, OpenMV Cam H7 Storage: MicroSD
Data source location	Institution: Dedan Kimathi University of Technology City/Town/Region: Nyeri Country: Kenya Latitude and longitude for collected samples/data: 0°23′26.0"S 36°57′43.7" E
Data accessibility	Repository Name: DSAIL-Porini: Annotated camera trap images of wildlife species from a conservancy in Kenya Data identification number: 10.17632/6mhrhn7rxc.6 Direct URL to data: https://data.mendeley.com/datasets/6mhrhn7rxc/6 The following GitHub repositories include all the code that was used to collect the data: https://github.com/DeKUT-DSAIL/cameratrap-pi.git https://github.com/DeKUT-DSAIL/powering-raspberrypi.git https://github.com/DeKUT-DSAIL/cameratrap-openmv.git

## Value of the Data

• This dataset contains RGB images of 6 mammalian species found in Central Kenya. It contains sex-specific annotations for 3 species and counts for all species which can be used to train and test computer vision-based wildlife censusing algorithms.
• The dataset is valuable for the field of Computer Vision, especially for the tasks of image classification and object detection.
• The dataset serves as an example of how high-quality ecological data can be acquired using low-cost and readily available hardware.
• The dataset can be used to test methodology to derive sex ratios and species distribution range maps from camera trap data. This information is valuable to ecologists.

## 1 Data Description

Camera traps have been extensively used in the documentation and study of wildlife species in areas of interest such as conservation reserves and national parks. They are attractive to ecologists because they allow non-intrusive data capture in the areas of interest. There are many examples of camera trap designs that have been utilized in recent projects including the Snapshot Serengeti camera survey commissioned to expand the monitoring of lions by providing continuous data on lions and other prey during the day and night [1]. The Tanzanian Wildlife Research Institute (TAWIRI) also used aerial photography to study herbivore populations [1] and recently, the 2021 Kenya National Wildlife Census was conducted to record wildlife census in the country's conservation area network [2].

This paper describes an annotated dataset of camera trap images collected at the Dedan Kimathi University Wildlife Conservancy (DeKUWC) in Kenya [3]. The data has six different species of mammals found in the conservancy. These mammalian species include impalas, Burchell's zebras, common warthogs, bushbucks, defassa waterbuck, and the Sykes’ monkey [3,4].

**Fig. 1:**
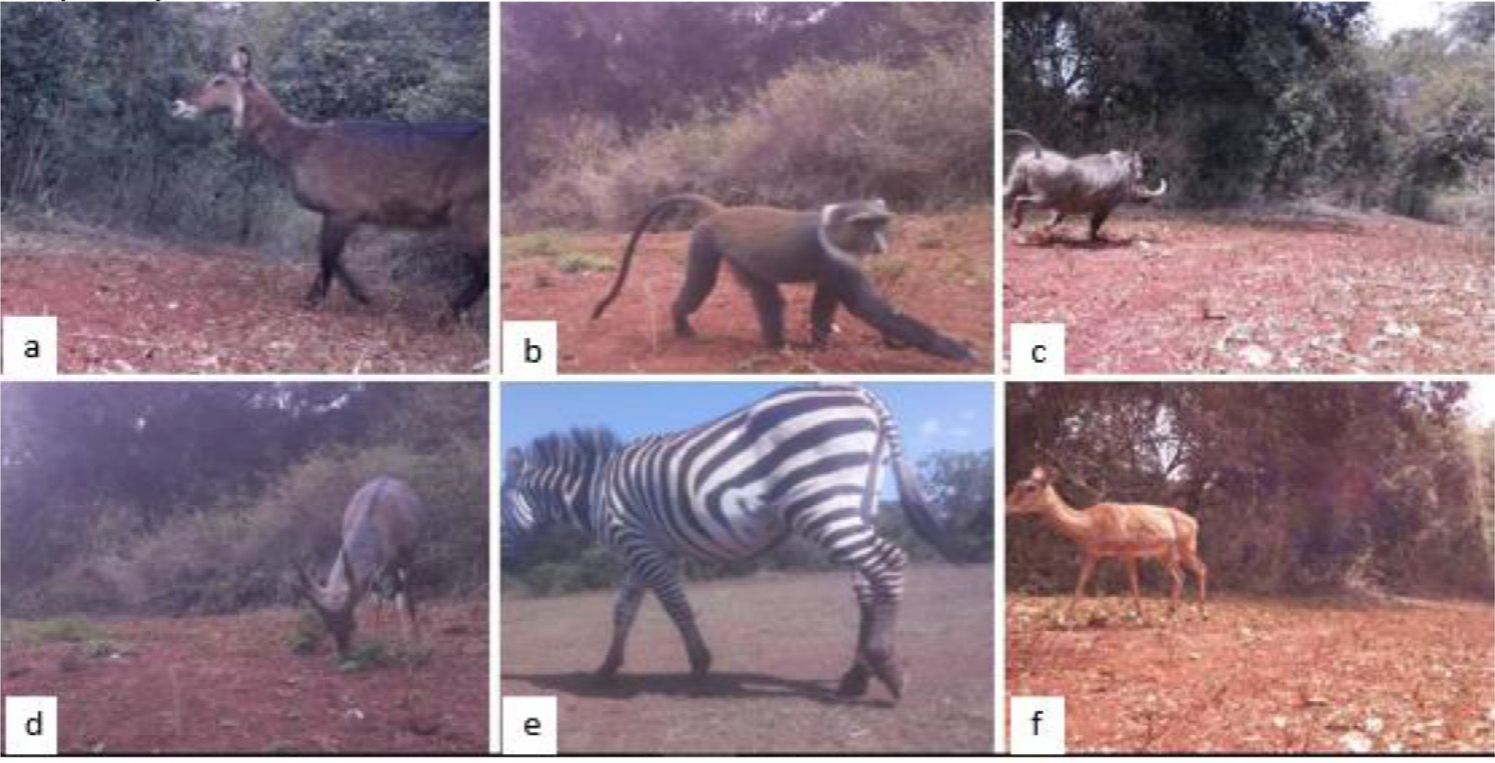
Animal species found in Dedan Kimathi University's Wildlife Conservancy: (a) defassa waterbuck, (b) Sykes monkey, (c) common warthog, (d) bushbuck, (e) Burchell's zebra, (f) impala [4].

The dataset consists of 8524 images from the four camera traps deployed. Two camera traps use the Raspberry Pi 2, one uses the Raspberry Pi Zero and the other uses the OpenMV Cam H7 [4]. 7589 images are from the Raspberry Pi 2, 610 images are from the Raspberry Pi Zero, and 325 images are from the OpenMV Cam H7 [4]. The images from the Raspberry Pi 2 and the Raspberry Pi Zero have an image size of 1280 × 720 pixels while the images from the OpenMV Cam H7 have a size of 640 × 480 pixels. All the images are in JPG format. Fig. 1 shows a sample of the images captured in the conservancy using the devices mentioned. The dataset provides an example of images captured using camera traps that can be fabricated by ecologists leveraging low-cost hardware platforms such as the Raspberry Pi.

From Table 1, the species captured the most was the impala. The species captured the least was the Sykes’ monkey. Due to factors such as the animal species being too far from the camera's angular field of view, animals covered by vegetation, or the presence of lens flare caused by the position of the camera relative to the incident sun rays, it was difficult to determine the species type in some of the images acquired.

**Table 1:** The number of images in the dataset with species in the conservancy. These are images captured by the Raspberry Pi 2, the Raspberry Pi Zero, and the OpenMV Cam H7 systems [4].

Species	Number of images containing the species
Bushbuck	313
Impala	5,649
Sykes’ monkey	52
Defassa waterbuck	606
Common warthog	1,565
Burchell's zebra	598

### 1.1 Data annotation

The DSAIL-Porini dataset was manually annotated with the following fields:

1. **Filename**

This is the name an image file was saved as. The filename takes the timestamp form of YY-MM-DD-H-M-S and contains the exact date and time it was taken and saved.

2. **Species**

**Fig. 2:**
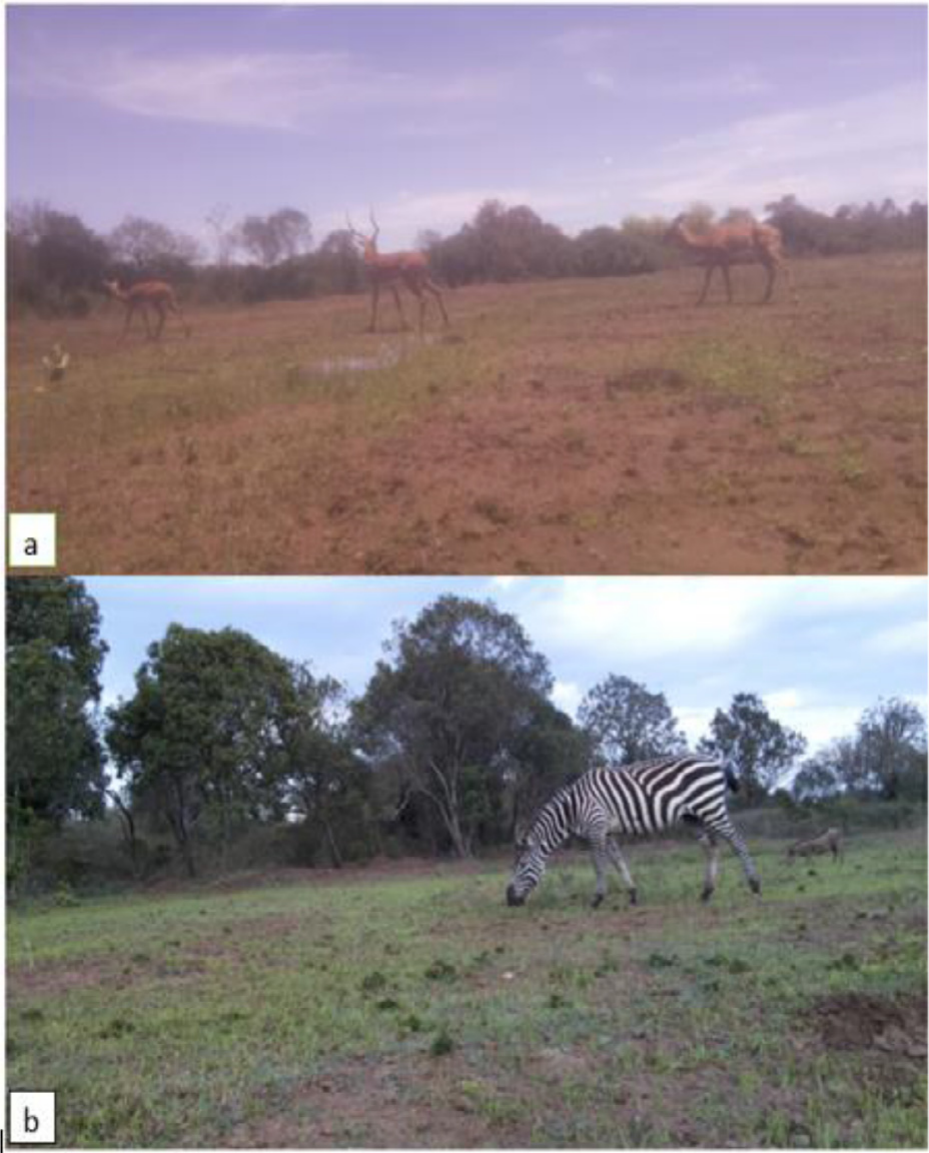
Examples of images from the dataset (a) In this image, we have three impalas. From the left: a female impala, a male impala (with horns), and another female impala. (b) In this image, we have a zebra and a warthog [4].

This is the species identified in each image. Some images contained only one species. For example, Fig. 2(a) contains only impalas. Other images had more than one species. For example, Fig. 2(b) has two species, a zebra and a warthog. Some images also had more than two species present. For the images with more than one or two species present, the order of labeling was from foreground to background and left to right. In Fig. 2(b), ‘zebra’ appears first in the species column because it appeared in the foreground and on the far left compared to the warthog.

3. **Count**

This column contains the number of individuals per species in a particular image. With this column, if there were more than one species in the preceding column, the order of the count column followed the order of the species column.

4. **Sex**

This column contains the sex of each animal in a particular image. With this column, the sex of the animals was labeled from left to right. Due to the lack of sex-revealing features with some animal species like the zebra and the warthogs, the sex column for these species was filled with the “Can't Tell” label. The impala has obvious features that enable the differentiation between a male and a female. The male impala commonly referred to as the impala ram has slender, lyre-shaped horns while the female impala commonly referred to as the impala ewe does not have horns. Similarly, only male bushbucks and waterbucks have horns.

5. **Latitude and Longitude**

These columns contain the coordinates of the location the camera trap was deployed at the time the image was captured.

Fig. 2 shows two examples of images from the dataset while Table 2 shows the corresponding annotation.

**Table 2:** This table shows the corresponding annotation of the images in Fig. 2[4].

	Filename	Device	Species	Count	Sex	Latitude	Longitude
a.	2022-09-28-12-53-14.jpg	Raspberry Pi 2	IMPALA	3	FEMALE, MALE, FEMALE	-0.390381	36.962386
b.	2022-10-12-17-33-47.jpg	Raspberry Pi 2	ZEBRA, WARTHOG	1,1	CAN'T TELL, CAN'T TELL	-0.390224	36.962036

In Table 2 row a, there is an image captured using the Raspberry Pi 2-based camera trap. The species present in this image is the impala and the number of impalas captured is 3. From the left, there is a female impala, a male impala, and another female impala. The last 2 columns are where the camera trap was deployed when the image was captured. In row b, there is another image captured using the Raspberry Pi 2-based camera trap. The species present in this image are the zebra and warthog. The number of zebras captured is 1 and the number of warthogs captured is 1. From the left, there is a zebra and a warthog whose sex can't be determined from this particular image. The last 2 columns are where the camera trap was deployed when the image was captured.

The data annotation is available on Mendeley Data as a .xlsx file. Images from the Raspberry Pi 2 and Raspberry Pi Zero are saved in one folder, ‘RaspberryPi_images’. In this folder, there are subfolders named in order of when the camera traps were deployed in the conservancy while images from the OpenMV Cam H7 are saved in another folder, ‘OpenMV_images’.

### 1.2 Count and sex distribution analysis

**Fig. 3:**
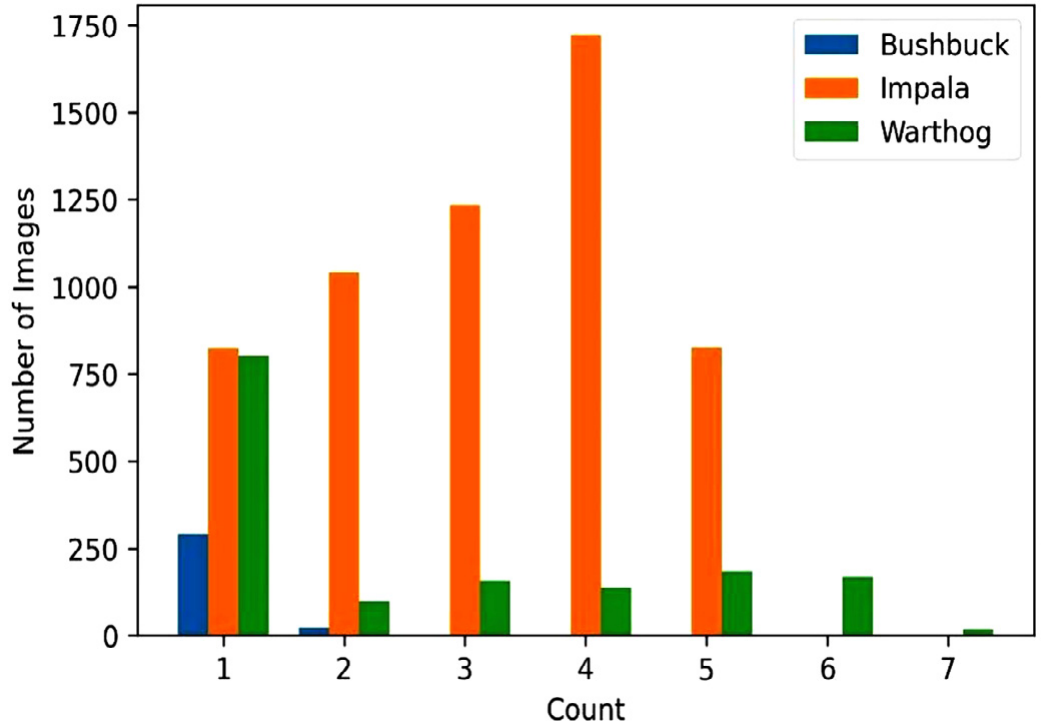
Count of animals in each image containing the bushbuck, impala, and common warthog species.

In the dataset released, count analysis was important because it aided in learning the behavioral patterns of some animal species in a controlled natural habitat and helped in keeping track of the species populations. From the images captured, some animals were mostly seen in groups while others were solitary. At times the animals would be feeding or just walking. Fig. 3 shows a count analysis of the images with the bushbuck, impala, and common warthog species. From the plot, the impalas are observed in groups ranging in size from one to five individuals with four individuals being the most frequent group size. Similarly, warthogs were captured in groups of up to seven individuals. In contrast, the bushbucks were mostly observed in isolation.

**Fig. 4:**
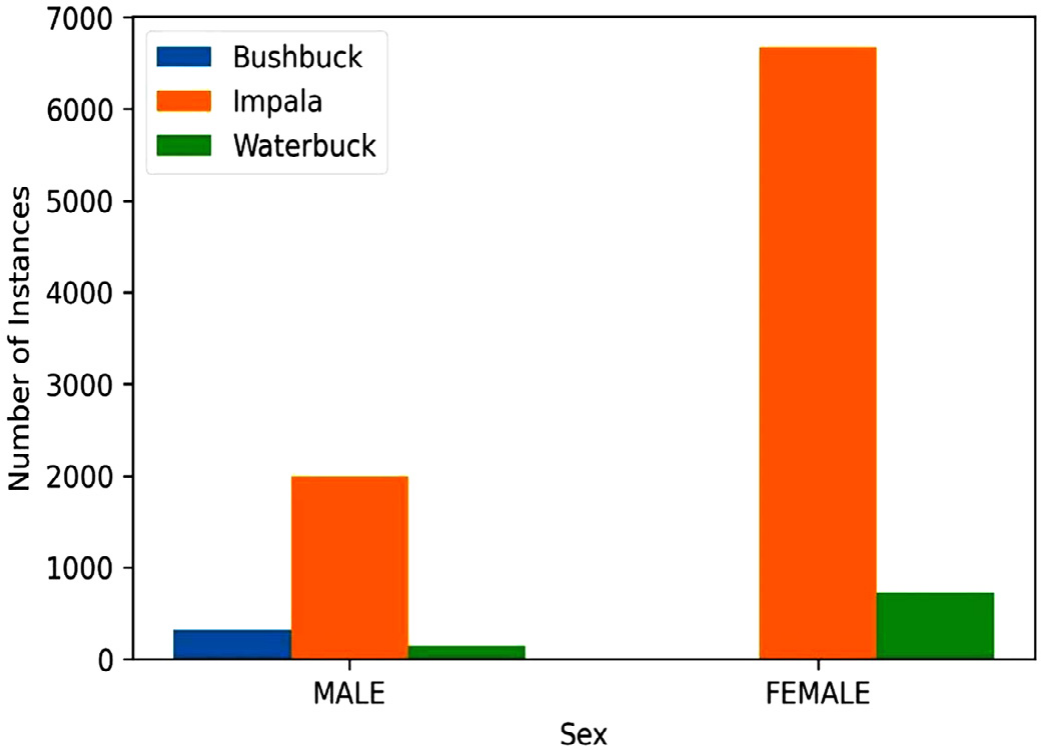
Sex distribution of the bushbuck, impala, and defassa waterbuck species.

Sex distribution analysis is also important because seeing the male and female species interact and knowing their distribution can help determine future population sizes. Fig. 4 shows the sex distribution of the bushbuck, impala, and waterbuck species. There are 1999 instances of Male impalas and 6672 instances of female impalas captured in the dataset. Interestingly there are more instances of male bushbucks than female bushbucks which can point to a male-skewed sex ratio.

## 2 Experimental Design, Materials and Methods

The following sections describe the methods used to acquire these data.

### 2.1 Hardware & software

Raspberry Pi-based and OpenMV Cam H7-based camera traps were designed and developed at DSAIL. The camera traps were also loaded with software to run them. The sections below describe the two camera traps.

#### 2.1.1 Raspberry Pi camera trap

**Fig. 5:**
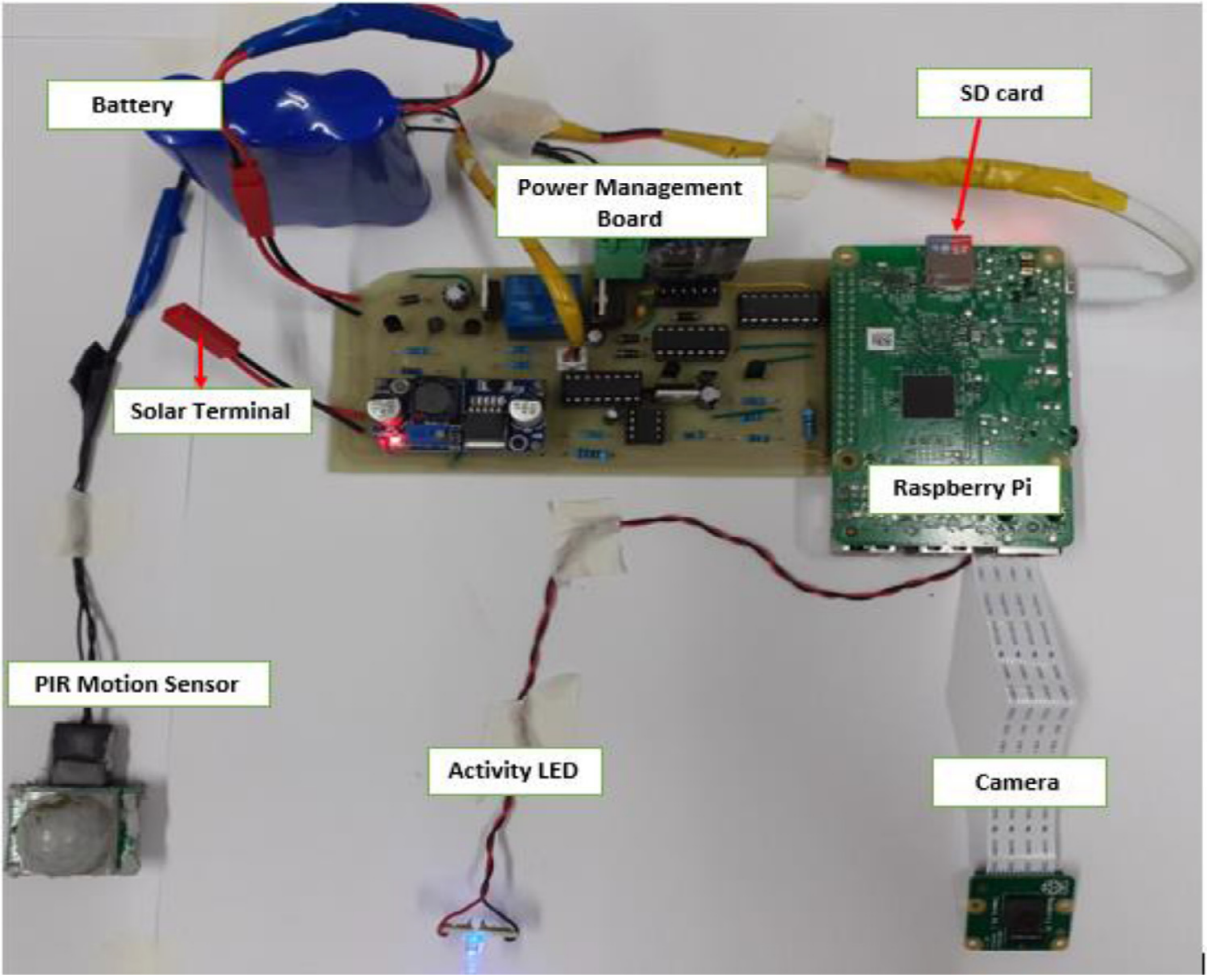
Hardware components of the Camera trap.

Three of our camera traps use Raspberry Pi as the setup control. It is used to capture the images, monitor battery voltage, and control the booting and shutting down of the system if the rated cut-off voltage of the battery is reached or if the time set for shutting down has been reached. To achieve this a power management board developed for an acoustic monitoring system shown in Fig. 5 is used. The board powers the Raspberry Pi autonomously giving it the ability to shut down safely when the battery gets drained or when it is scheduled to shut down and wakes up later when the battery gets charged or at the scheduled wake-up time [5,6].

Once the Passive Infrared (PIR) sensor is triggered, an image is captured and saved in the micro-SD card. The program has a 2-second delay meaning it will wait 2 seconds before capturing another image even if the sensor is triggered. The captured images are saved with the format YYYY-MM-DD-H-M-S, to help keep track of the exact time the image was captured.

**Fig. 6:**
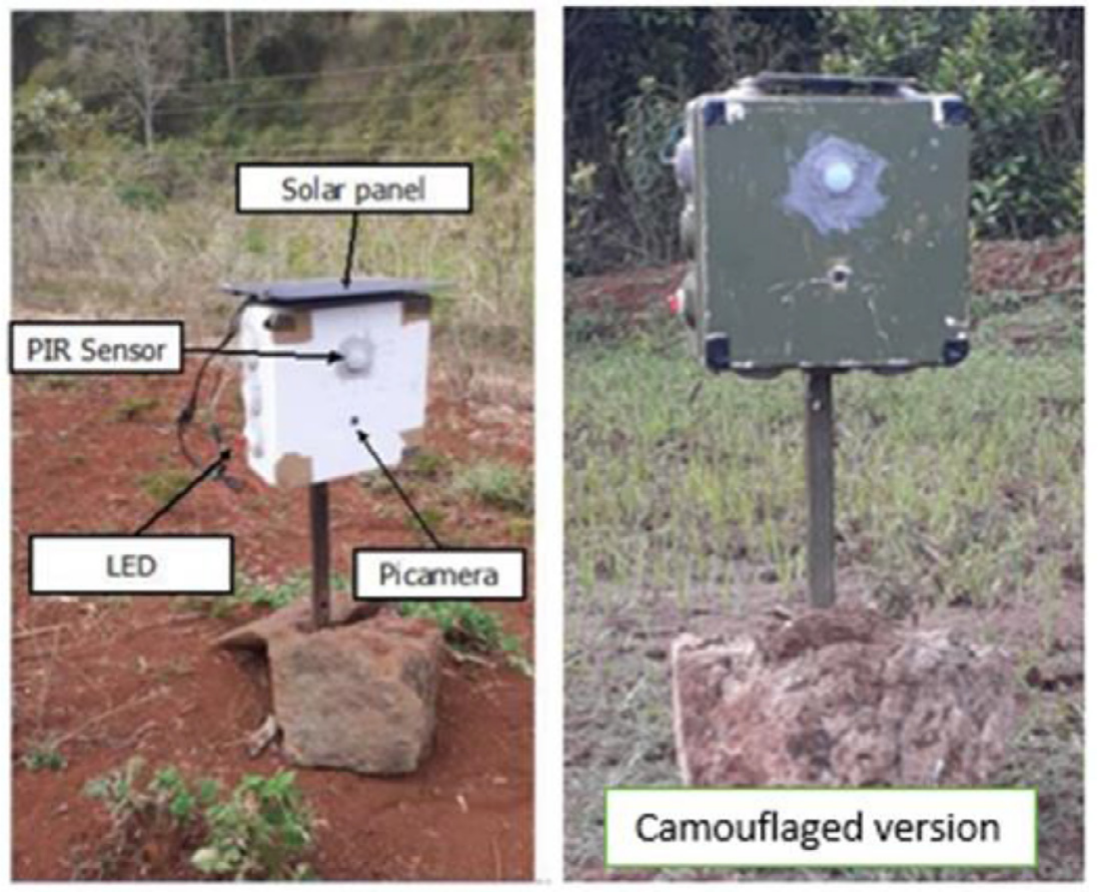
The Camera trap in deployment. (a) Before painting (b) After painting.

All hardware was housed in a waterproof enclosure box and deployed in the wildlife conservancy. The camera traps were painted green to camouflage with the surrounding, as shown in Fig. 6. There was a noticeable change after painting, and better quality images of animals, especially waterbucks and zebras, were captured.

#### 2.1.2 OpenMV Cam H7 camera trap

The OpenMV Cam H7 is a relatively small low-power development board with a micro-SD card socket. It has a powerful onboard 5MP camera and it can be programmed in Python. It is used to run computer vision algorithms on what the OpenMV Cam H7 captures for areas like detection and color tracking. Other features are the ability to capture grayscale/RGB images/videos, low power consumption while processing images, and the ability to track color blobs [7]. The OpenMV Cam H7 camera trap captures images once a motion sensor is triggered.

### 2.2 Camera trap placement

**Fig. 7:**
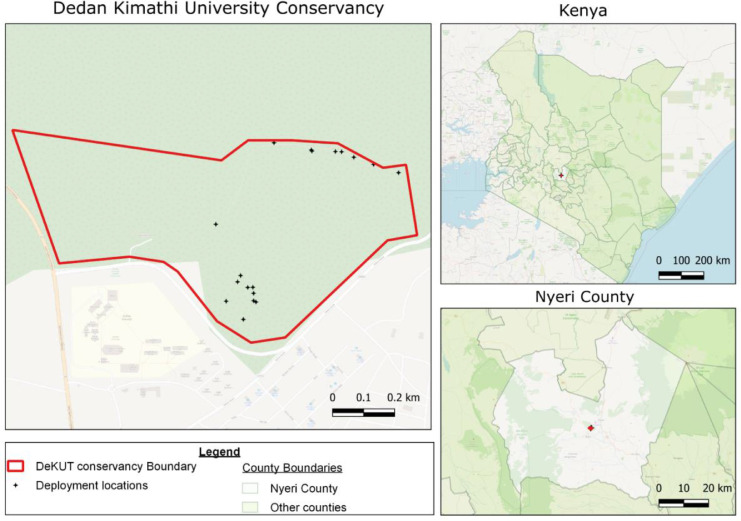
(a) Kenyan map showing the location of Nyeri County. (b) A map showing the conservancy within the county. (c) Different deployment locations in the conservancy.

The camera traps mentioned were deployed at different points in the conservancy. Fig. 7 shows different parts of the conservancy where the camera traps have been deployed for image data collection.

The decision to place the traps at these locations was informed by factors such as the location of the mineral supplements provided for the animals, paths leading to water points, areas around water points, areas of interest pointed out by the wardens, paths leading to animal resting points and location of grazing areas.

A total of 30 deployments have been made at 18 different locations in the conservancy. During a deployment window, the camera traps take shots and store the images on SD cards. After about a week, the camera traps are retrieved and the images are offloaded to a computer for annotation. The camera traps are then redeployed for data collection.

## Ethics Statements

It does not apply to this dataset.

## Funding

This work was funded by Data Science Africa through the Centre for Data Science and Artificial Intelligence (DSAIL) program, Canada's International Development Research Centre (IDRC), and the Swedish International Development Cooperation Agency (Sida) through the Artificial Intelligence for Development in Africa (AI4D Africa) program. We also thank Google for a research award to DSAIL.

## CRediT authorship contribution statement

**Lorna Mugambi:** Software, Data curation, Writing – original draft. **Jason N. Kabi:** Software, Writing – review & editing. **Gabriel Kiarie:** Software, Writing – review & editing. **Ciira wa Maina:** Conceptualization, Writing – review & editing, Supervision.

## Declaration of Competing Interest

The authors declare that they have no known competing financial interests or personal relationships that could have appeared to influence the work reported in this paper.

## Data Availability

DSAIL-Porini: Annotated camera trap images of wildlife species from a conservancy in Kenya (Original data) (Mendeley Data).
